# Successful Salvage Radiotherapy for a Chemo-refractory, Non-resectable, Undifferentiated Pleomorphic Sarcoma Lung Metastasis with Pericardial Involvement: A Case Report

**DOI:** 10.7759/cureus.445

**Published:** 2016-01-02

**Authors:** Thuraya Al-Hajri, Jessica Chan, Jean-Michel Caudrelier

**Affiliations:** 1 Radiation Oncology, The Ottawa Hospital Cancer Centre, The University of Ottawa

**Keywords:** sarcoma, imrt, lung mets, pericardial

## Abstract

We report a case of an undifferentiated pleomorphic sarcoma in a 73-year-old female, with a solitary lung metastasis involving the pericardium that progressed on first-line chemotherapy. Partial removal of the lesion was achieved after lingular segmentectomy, which required en-bloc pericardial resection due to deep pericardial invasion. However, the residual disease significantly grew despite second-line chemotherapy, and the tumor became unresectable due to near encasement of the left anterior descending coronary artery. Therefore, we opted for a salvage radical dose of intensity-modulated radiotherapy (60Gy in 25 fractions) to the pericardial lesion. No acute side effects were observed, and after three years of follow-up, good local control has been achieved with no significant late effects observed. This case suggests that radical radiotherapy using IMRT could be considered to treat sarcomatous pericardial lesions in patients who do not respond to chemotherapy and who are inoperable or non-resectable.

## Introduction

Undifferentiated pleomorphic sarcomas (UPS) account for less than 5% of adult soft-tissue sarcomas (STS) [1]. However, they are the most common STS seen in patients over age 40. UPS typically progress and enlarge rapidly, with an overall 5-year survival rate of 50–60%. Approximately 5% of UPS patients have metastases at initial presentation, most commonly to the lung [2].

Pulmonary metastases located near or involving the heart pose a particular challenge to radiation oncologists due to cardiac-dose limitations, which account for potential radiation-induced cardiotoxicity.  Advanced techniques, such as intensity-modulated radiotherapy (IMRT) offer improved tumor-dose conformity and normal tissue sparing, but limited information is available on its effectiveness for treating metastases with pericardial involvement from STS. Here, we report a case in which a radical dose of radiotherapy using IMRT produced excellent tumor control without acute or long-term treatment-related side effects for a chemo-refractory, non-resectable, sarcomatous pulmonary metastasis with pericardial involvement. 

## Case presentation

In 2009, a 73-year-old female noticed a painful left thigh mass that increased in size over 6 months, causing functional impairment. On exam, an indurated and hard mass was palpable in her medial left thigh, measuring 10 cm craniocaudally. Subsequent MRI showed a large heterogeneously enhancing mass in the vastus medialis muscle extending into the vastus intermedius, consistent with a sarcomatous lesion. Core biopsies confirmed high-grade UPS. Staging CT images were negative for metastatic disease. She received preoperative radiotherapy to the tumor volume, 50 Gy in 25 fractions, followed by surgical resection one month later. Pathology confirmed UPS measuring 12x8x7 cm and showing 95% necrosis. Resection margins were negative. The patient declined adjuvant chemotherapy.

**Figure 1:**
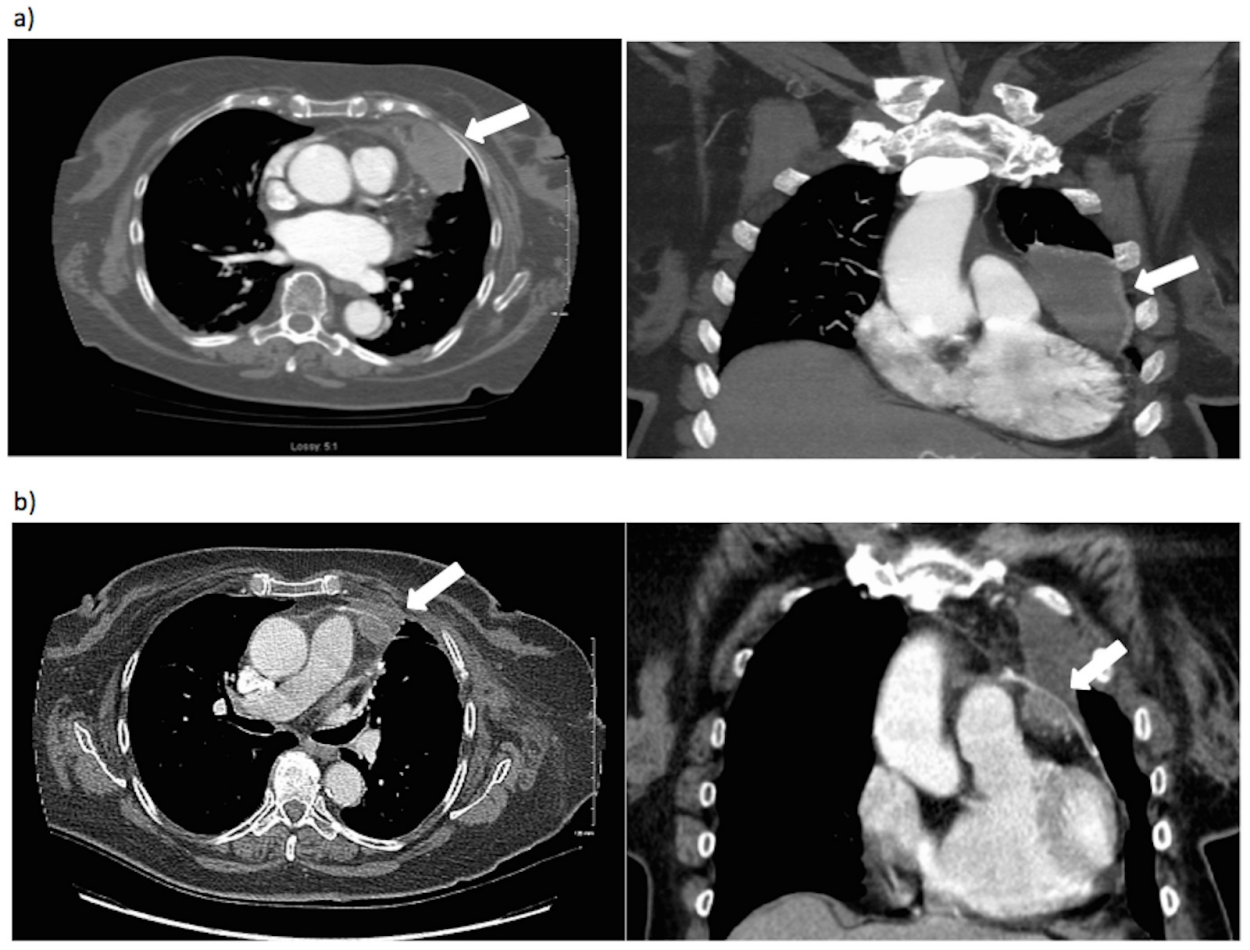
Axial and coronal computed tomography scans of the lesion (a) Pre-surgical resection, July 2011; (b) Post-surgical resection, September 2011.

In July 2010, four months post-surgery, a follow-up CT chest showed two new dominant, well-circumscribed pulmonary nodules consistent with metastases. All other imaging investigations were negative. Surgical resection was initially planned; however, a pre-operative repeat CT thorax showed an increasing number of pulmonary nodules. The patient was no longer a candidate for surgery, and she proceeded to receive six cycles of palliative doxorubicin. All of her lung nodules resolved, but one left upper lobe nodule increased (0.9 cm to 2.8 cm, maximum diameter). Needle biopsy of this lesion showed predominantly necrosis. Surgery was again planned to resect this isolated focus of metastasis. However, in July 2011 while waiting for surgery, the patient presented to the emergency department with retrosternal chest pain. A CT chest showed dramatic growth of her left upper lobe lesion, now measuring 6x7 cm, sitting adjacent to the anterior pericardium (Figure 1a). Three weeks later, a lingular segmentectomy was performed. Intraoperatively, there was evidence of deep pericardial invasion requiring en bloc pericardial resection. Pathology confirmed a positive deep margin with UPS present on the inner pericardial surface, with negative lateral pericardial and lung parenchymal resection margins. A small, biopsy-proven area of metastasis on the epicardial fat near the left anterior descending coronary artery (LAD) was also found intraoperatively, but could not be resected due to its close LAD proximity.

**Figure 2:**
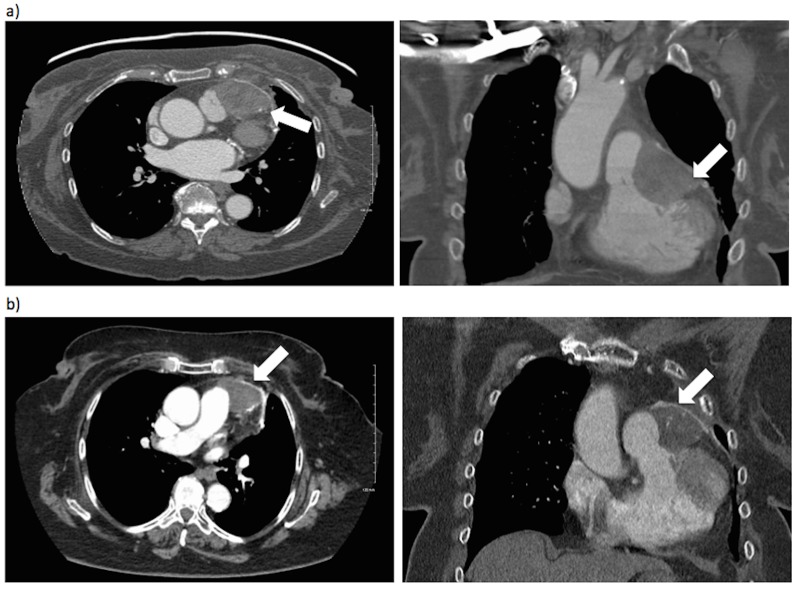
Axial and coronal computed tomography scans of the lesion (a) Pre-salvage radiotherapy, June 2012; (b) Post-salvage radiotherapy in follow-up, June 2015.

The postoperative CT showed a residual 2.7x1.4 cm soft tissue density in the left anterior mediastinum adjacent to the anterior pericardial line, consistent with residual tumor (Figure 1b). Palliative gemcitabine/docetaxel was started in October 2011. Unfortunately, over eight months of chemotherapy, there was clear tumor progression. In June 2012, the mass grew up to 5.6x3.8x5 cm (Figure 2a). It extended from the inner surface of the pericardial patch causing compression over the right ventricular outflow tract and main pulmonary artery with subsequent enlargement of the right ventricle and atrium. In addition, the mass appeared to encase the mid-LAD. A 3.8x1.4x6.5 cm soft-tissue mass was also seen extending to the outer surface of the pericardial patch towards the chest wall. Additional CT scans showed no other thoracic or intra-abdominal metastases. Cardiac surgery was consulted, but concluded the lesion was non-resectable given its close proximity to and involvement of the LAD. The patient was referred to radiation oncology, and given her good performance status and the lack of other uncontrolled systemic disease, we opted for a radical dose of radiotherapy.

**Figure 3:**
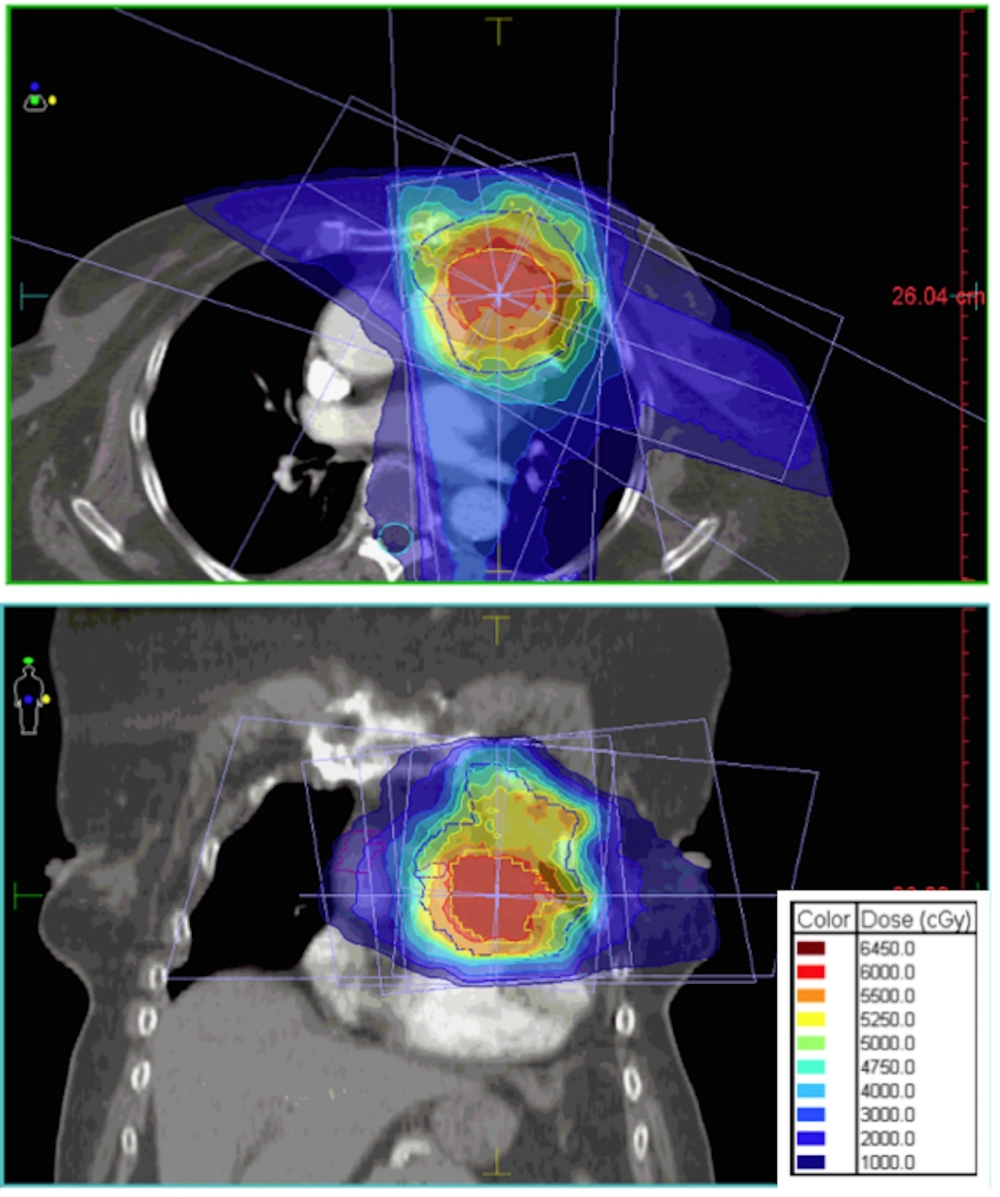
Isodoses from IMRT planning on axial and coronal views.

A 4D-CT chest simulation with contrast was completed with the patient in supine position over a lung board. The gross tumor volume (GTV) was the mass in free-breathing CT, and the GTV maximum intensity projection (MIP) was the GTV visualized on the 4D-CT images set, using all phases of respiration as done routinely for most lung stereotactic body radiation treatments. Both GTV and GTV MIP were combined to create the internal treatment volume (ITV). One-centimeter margins were added to the ITV to create the clinical target volume (CTV), with an additional 5-millimeter margin added for the planning target volume (PTV). The PTV was prescribed in a dose of 50 Gy in 25 fractions of 2 Gy, and the ITV concomitantly received a dose of 60 Gy in 25 fractions of 2.4 Gy. IMRT step-and-shoot technique using 5 coplanar beams spaced from 25 to 120 degrees and covering a sector from 25 to 350 degrees were defined. Dose calculations were done on the MONACO treatment planning system using 6MV photons and the Monte Carlo algorithm. The organs at risk were the heart and cardiac subunits (Figure 3). Based on the dose volume histogram, 96% of the PTV was covered by 50 Gy, and 84% of the ITV was covered by 60 Gy. The mean dose to the main and distal LAD was 54 Gy and 42 Gy, respectively. Mean heart dose was 15 Gy, with a V40 of 11.6%. The V20 and V5 of both lungs were 15.5% and 33%, respectively. Cone-beam CT was used for daily positioning verification. The radiation treatment was completed in August 2012 and was extremely well tolerated without any acute side effects during or immediately following treatment.

 

The patient continued to show no signs or symptoms of moderate-to-late radiation-induced side effects for three years post-radiotherapy, with the patient last seen in-clinic in November 2015. Follow-up CTs during this time also showed a stable pericardial lesion with no progression or disease recurrence, including the most-recent CT in July 2015 (Figure 2b). 

## Discussion

This case highlights the safe and long-term tumor control achieved by using salvage IMRT for a chemotherapy-resistant, non-resectable sarcomatous lung metastasis with pericardial involvement. Generally, chemotherapy and/or surgery are first considered in the treatment of pulmonary metastases from STS--depending on the number of lesions, site, and disease-free interval time [3]. Unfortunately, our patient failed second-line chemotherapy post-incomplete surgical resection, and re-resection was not indicated, leaving radiotherapy as the only local treatment option.

The location of the tumor within the pericardium was challenging due to the possible acute and long-term radiation-induced cardiac side effects including pericarditis, angina and myocardial infarction [4]. In addition, the correlation between the radiation dose volume and most cardiac toxicities is not yet clear [5]. There is also still only a limited amount of data on the success of radiotherapy in treating cardiac STS metastases, with some suggesting a total dose of at least 45 Gy for such cases [6].

Despite this, the role of radiotherapy in managing advanced STS has increased with recent technological advancements, such as IMRT’s ability to improve tumor-dose conformity and normal tissue sparing [7]. We applied the advantages of IMRT to our patient’s challenging case, which likely led to her exceptional response given our ability to deliver a conformal radical dose of radiation with concomitant dose boost within the GTV, while sparing a relatively large proportion of the surrounding normal cardiac and lung structures. In addition, we opted for a standard radical dose of 60 Gy in 25 fractions to allow for the best chance of tumor control. It is possible that a lower dose using a hypofractionation-radiation regimen could have produced similar outcomes. However, given the rarity of this clinical case presentation, it is difficult to evaluate the efficacy of this alternative approach. 

## Conclusions

Our case report suggests that high-dose radiotherapy using IMRT is a feasible treatment for UPS lung metastases involving the pericardium and may provide excellent tumor control without significant radiation-induced side effects. 
